# Smoking, physical inactivity and obesity as predictors of healthy and disease-free life expectancy between ages 50 and 75: a multicohort study

**DOI:** 10.1093/ije/dyw126

**Published:** 2016-08-02

**Authors:** Sari Stenholm, Jenny Head, Mika Kivimäki, Ichiro Kawachi, Ville Aalto, Marie Zins, Marcel Goldberg, Paola Zaninotto, Linda Magnuson Hanson, Hugo Westerlund, Jussi Vahtera

**Affiliations:** 1 Department of Public Health, University of Turku, Turku, Finland; 2 National Institute for Health and Welfare, Helsinki, Finland; 3 Department of Epidemiology and Public Health, University College London, London, UK; 4 Clinicum, Faculty of Medicine, University of Helsinki, Helsinki, Finland; 5 Department of Social & Behavioural Sciences, Harvard T.H. Chan School of Public Health, Boston, MA, USA; 6 Finnish Institute of Occupational Health, Turku, Finland; 7 Population-based Epidemiologic Cohorts Unit-UMS 011, F-94807, Villejuif, France; 8 Versailles St-Quentin Univ, UMS 011, F-94807, Villejuif, France; 9 Aging and Chronic Diseases, Epidemiological and Public Health Approaches, U 1168, Villejuif, France; 10 Stress Research Institute, Stockholm University, Stockholm, Sweden and; 11 Turku University Hospital, Turku, Finland

**Keywords:** healthy life expectancy, obesity, physical inactivity, smoking, cohort study

## Abstract

**Background:** Smoking, physical inactivity and obesity are modifiable risk factors for morbidity and mortality. The aim of this study was to examine the extent to which the co-occurrence of these behaviour-related risk factors predict healthy life expectancy and chronic disease-free life expectancy in four European cohort studies.

**Methods:** Data were drawn from repeated waves of four cohort studies in England, Finland, France and Sweden. Smoking status, physical inactivity and obesity (body mass index ≥30 kg/m^2^) were examined separately and in combination. Health expectancy was estimated by using two health indicators: suboptimal self-rated health and having a chronic disease (cardiovascular disease, cancer, respiratory disease and diabetes). Multistate life table models were used to estimate sex-specific healthy life expectancy and chronic disease-free life expectancy from ages 50 to 75 years.

**Results:** Compared with men and women with at least two behaviour-related risk factors, those with no behaviour-related risk factors could expect to live on average8 years longer in good health and 6 years longer free of chronic diseases between ages 50 and 75. Having any single risk factor was also associated with reduction in healthy years. No consistent differences between cohorts were observed.

**Conclusions:** Data from four European countries show that persons with individual and co-occurring behaviour-related risk factors have shorter healthy life expectancy and shorter chronic disease-free life expectancy. Population level reductions in smoking, physical inactivity and obesity could increase life-years lived in good health.

Key messages

- Multistate life table models were used to estimate healthy life expectancies between ages of 50 and 75 in four European cohort studies.
- Non-smoking, physically active and non-obese men and women lived on average 8 years longer in good health and 6 years longer free of chronic diseases between ages 50 and 75, compared with those with at least two behaviour-related risk factors.
- Of the individual behaviour-related risk factors, physical inactivity was associated with the greatest reduction in healthy years and obesity with greatest reduction in chronic disease-free years.
- Our results support the view that reducing smoking, physical inactivity and obesity could substantially increase the time spent in good health in the population.

## Introduction

The world's population is ageing at a rapid pace. Rising life expectancy (LE) represents one of the major human success stories,^1^ but not all the increased years of life are being spent in optimal health. A recent Global Burden of Disease study suggests that the increases in healthy, disease-free years has not been as large as the growth in LE; as a result, people are living more years with illness and disability.^2^ Estimation of health expectancy provides a single summary measure of a population's health, which takes into account morbidity and mortality, and is therefore useful when comparing the health in different populations and population sub-groups.^3^^,^^4^

A study based on data from 11 European countries estimated that 60% of deaths from all causes could be attributed to behaviour-related risk factors.^5^ Furthermore, the importance of health behaviours for the prevention of chronic diseases, such as type 2 diabetes, coronary heart disease and cancer, is widely acknowledged. Smoking, physical inactivity and obesity are among the top 10 behaviour-related risk factors for burden of diseases in developed countries,^6^ and they have also been shown to be associated with shorter health expectancy and LE.^7––9^. The cumulative impact of multiple behaviour-related risk factors on health expectancy is of interest because studies show that people who engage in multiple risk behaviours have higher mortality,^10–14^ increased risk of chronic diseases^15–17^ and poor cognitive^18^ and lower physical functioning^19^ compared with people who have no or only one behaviour-related risk factor.

Previous studies have estimated healthy years and disability-free years separately for smoking and obesity.^20^^,^^21^ In addition, there are at least two large studies that used information on past trends or current levels of obesity and smoking to estimate the combined effect of obesity and smoking on quality-adjusted LE and disability-free LE.^22^^,^^23^ Of the two risk factors, obesity appeared to be the main driver for shortened disability-free LE. However, neither of these studies considered low physical activity among the risk factors.^24^ This is a limitation, as regular physical activity is known to be associated with reduced risk of several chronic diseases,^25^^,^^26^ better physical and cognitive functioning in old age and higher longevity.^27–29^

To address some of these limitations, we examined the extent to which the co-occurrence of three modifiable behaviour-related risk factors, namely smoking, physical inactivity and obesity, predicted healthy LE and chronic disease- free LE in a large dataset of older men and women in England, Finland, France and Sweden. In addition, we estimated the associations of individual risk factors with these outcomes.

## Methods

### Study population

We used data from four prospective cohort studies from England, Finland, France and Sweden to calculate partial LE and health expectancies between the ages of 50 and 75. In all cohorts, people aged 50 years or older with valid data on health and behaviour-related risk factors were included from the first observation. We limited our estimation of partial LE to an upper age of 75 as not all cohorts had participants aged 75 and older, and this choice allowed us to have a comparable time frame for each cohort.

The English data are from the first six waves of the English Longitudinal Study of Ageing (ELSA), an open-access, nationally representative biennial longitudinal survey of those aged 50 and over living in private households in England. The sample size was 11 391 people at the first wave in 2002–03.^30^ We included 8805 participants aged 50 to 75 at baseline, who had valid measures of all three behaviour-related risk factors. For body mass index we used data collected during the nurse visit at either wave 2 or wave 0.

The Finnish data are from five waves of the Finnish Public Sector study (FPS). The FPS, established in 1997/98, comprises all 151 901 employees with a ≥6-month job contract in any year from 1991/2000 to 2005 in 10 towns and five hospital districts in Finland. Survey data have been collected by repeated surveys in 4-year intervals on all 103 866 cohort members, who were at work in the participating organizations during the surveys in the years 1997/98, 2000/01, 2004/05, 2008/09 and/or 2012/13. Follow-up survey data of the respondents who had retired or left the organizations were collected in 2005, 2009 and 2013. Of those, 8 848 participants responded at least once (response rate 82%). For the analysis, we used data from 42 516 participants aged 50 to 75 at the first wave for which valid data on all health behaviour-related risk factors were recorded.

The French data are from the GAZEL Cohort Study, established in 1989 among Électricité de France-Gaz de France (EDF-GDF) workers, the French national utility company, with annual waves of data collection up to 2014. It is a cohort characterized by a broad coverage of health problems and determinants. At inception in 1989, the GAZEL Cohort Study included 20 625 participants (1 011 men and 5614 women) working at EDF-GDF and then aged from 35 to 50 years. The cohort is broadly diverse in terms of social, economic and occupational status, health and health-related behaviours.^31^ We included participants who had valid measures of behaviour-related risk factors in 1996 (or a later year if missing risk factor data), as physical inactivity was measured for the first time in 1996. For the analysis we used data on 14 931 participants.

The data for Sweden came from five waves of the Swedish Longitudinal Occupational Survey of Health (SLOSH).^32^ The first wave of SLOSH in 2006 was a postal questionnaire follow-up of all respondents to the 2003 Swedish Work Environment Survey (SWES), a cross sectional, biennial survey of a random stratified sample of those gainfully employed people aged 16–64 years. At wave 2 in 2008 the sample was increased by adding the respondents from the 2005 SWES. The people were then re-surveyed in 2010, 2012 and 2014. A subsample from SWES 2007 was also followed up in 2010, while all participants from SWES 2007 and participants in SWES 2009 and 2011 were followed up in 2014. This yielded an overall sample of 40 877 women and men originally representative of the working population in Sweden in 2003-2011 of which 65% responded to a follow-up questionnaire at least once. The analytic sample in the present study comprised 8 118 participants who were aged 50 to 75 at the first wave for which valid data on all behavior-related risk factors was recorded.

In all cohorts, participants provided their informed consent to taking part. Ethical approval was obtained in each of the countries from relevant ethical committees/boards

### Measurement of behaviour-related risk factors

Tobacco smoking and physical inactivity were ascertained by using participant-completed questionnaires in FPS, GAZEL and SLOSH and by the interviewer in ELSA. Smoking status was dichotomized into current smokers vs former or never smokers.^33^ Leisure-time physical inactivity was defined as no or very little moderate or vigorous leisure-time physical activity or exercise vs regular physical activity.^34^ Body mass index (BMI) was calculated using self-reported body weight and height in FPS, GAZEL and SLOSH. In ELSA, body weight and height were measured by a study nurse in the participants’ homes. Obesity was defined as BMI ≥ 30 kg/m^2^.^35^Supplementary Data (available as Supplementary Data at *IJE* online) shows operationalization of behaviour-related risk factors in each cohort. Co-occurrence of behaviour-related risk factors (smoking, physical inactivity and obesity) was calculated as a sum of these risk factors and classified as 0, 1 and 2 or more risk factors.

### Outcome measures

In each study cohort, we defined two health expectancy outcomes: (i) healthy LE using suboptimal self-rated health; and (ii) chronic disease-free LE using occurrence of chronic diseases. In addition, we took into account mortality.

#### 

##### Self-rated health

All participants were asked about their health status at each wave. Responses were categorized into good and suboptimal health. In ELSA, FPS and SLOSH, participants were asked to rate their general health on a 5-point Likert scale, which was dichotomized by categorizing response scores 1–2 as good health and scores 3–5 as suboptimal health. GAZEL used an 8-point Likert scale (1 = very good, 8 = very poor), which was dichotomized by categorizing response scores 1–4 as good health and scores 5–8 as suboptimal health, as previously validated.^36^ Health expectancy based on self-rated health is labelled hereafter as healthy LE.

##### Chronic diseases

Presence of the following chronic diseases was ascertained in each study by asking ‘Has a doctor ever told you that you have…’: (i) heart disease (heart attack, coronary heart disease, angina, congestive heart failure, or other heart problems); (ii) stroke (stroke or transient ischaemic attack); (iii) chronic lung disease (chronic bronchitis or emphysema or asthma); (iv) cancer (cancer or a malignant tumour of any kind except skin cancer); and (v) diabetes (diabetes or high blood sugar). Individuals were defined as having a chronic disease if they reported one or more of these conditions. The presence of chronic diseases at baseline (first observation included in analysis) included any chronic diseases reported before the age of 50 from available information on respondents. Health expectancy based on chronic diseases is hereafter labelled as chronic disease-free LE.

##### Mortality

This was ascertained from linked register data for each study cohort with follow-up censored on 31 December of the year in which data collection last took place for each study cohort.

### Statistical analyses

Characteristics of the participating cohorts are presented at the first observation point, which refers to the date each participant is for the first time included in the dataset.

We applied multistate models to longitudinal data to obtain transition probabilities between health states. Discrete-time multistate life table models were used to estimate partial LE and healthy LE and chronic disease-free LE between the ages of 50 and 75 (in total 26 years). For both measures, three health states were defined: healthy, unhealthy and dead. For healthy LE, there were four possible transitions between the health states, namely: healthy to unhealthy (onset), unhealthy to healthy (recovery), healthy to dead and unhealthy to dead. For chronic disease-free LE, there were only three possible transitions as, by definition, recovery was not possible.

For each study cohort, age-specific transition probabilities by sex and combined behaviour-related risk factors were estimated from multinomial logistic models with age (in years), sex and socioeconomic position as covariates. Partial LE, healthy LE and chronic disease-free LE from ages 50 to 75 were then calculated based on these estimated transition probabilities using a stochastic (micro-simulation) approach.^37^ For each study, individual trajectories for a simulated cohort of 10 000 persons were generated with distributions of covariates at the starting point based on the observed study-specific prevalence by 5-year age group, sex, socioeconomic position and behaviour-related risk factors. Partial LE, healthy LE and chronic disease-free LE from age 50 to 75 were then calculated as the average from these trajectories for combined behaviour-related risk factors and sex. Computation of 95% confidence intervals (CI) (from 2.5th and 97.5th percentiles) for these multistate life table estimates was performed using a bootstrap method with 500 replicates for the whole analysis process (multinomial analysis and simulation steps). In addition, we repeated the analyses for each of the three behaviour-related risk factors separately. The analyses for individual behaviour-related risk factors and sensitivity analyses were conducted using a bootstrap method with 50 replicates. As behaviour-related transitions to poor health and death may differ by sex, we repeated analyses including interactions between sex and combined behaviour-related risk factors as well as each individual risk factors in the multinomial logistic models.

All analyses were conducted in SAS 9.2 using the SPACE (Stochastic Population Analysis of Complex Events) program [http://www.cdc.gov/nchs/data_access/space.htm].^38^ This program uses the stochastic (i.e. micro-simulation) approach to estimate the healthy LE as opposed to another well-known program, IMaCh (Interpolation of Markov Chains) which uses a deterministic approach.^39^

## Results

Characteristics of the study cohorts for men and women at the first observation point are shown in Table 1. Prevalence of suboptimal self-rated health varied across cohorts and ranged among men from 19% (GAZEL) to 37% (FPS); among women this prevalence varied between 21% (SLOSH) and 34% (FPS). Chronic diseases were most common among ELSA men (34%) and women (31%) and least common in SLOSH men (22%) and women (17%). At the first observation point, about half of the ELSA, FPS and GAZEL participants and 60% of SLOSH participants were free of all three behaviour-related risk factors. One in 10 individuals had two or three behavioural-related risk factors in all cohorts. The most common behaviour-related risk factors were obesity in ELSA and physical inactivity in FPS, GAZEL and SLOSH.

**Table 1.** Characteristics of the contributing cohorts at the first observation point^a^

	ELSA		FPS		GAZEL		SLOSH	
	Men	Women	Men	Women	Men	Women	Men	Women
Sample size	4072	4733	8343	34173	11098	3833	3663	4326
Age (mean, SD)	61.51 (7.20)	61.42 (7.29)	53.59 (3.16)	53.15 (2.92)	51.97 (2.20)	51.35 (2.05)	57.95 (5.76)	57.32 (5.67)
Socioeconomic position (%)								
High grade	36.49	24.93	42.04	27.04	32.83	9.24	22.76	17.08
Middle grade	19.77	27.32	24.06	56.37	55.33	67.65	36.50	51.66
Low grade	43.74	47.75	33.91	16.59	11.84	23.12	40.74	31.26
Suboptimal self-rated health (%)	25.15	23.83	37.25	34.09	19.17	23.51	23.85	20.52
Chronic diseases (%)^b^	34.39	31.29	25.52	26.00	23.09	25.31	21.68	17.22
Co-occurrence of behaviour-related risk factors (%)								
0	55.67	49.48	53.57	59.96	49.36	47.30	60.35	64.95
1	34.36	37.46	32.79	30.06	37.89	41.12	29.59	27.29
≥2	9.97	13.06	13.64	9.98	12.75	11.58	10.06	7.76
Smoking, %	19.13	20.45	21.62	15.31	19.57	14.27	15.37	18.48
Physical inactivity, %	11.96	15.28	24.51	21.32	37.40	43.49	21.02	12.51
Obesity, %	23.99	28.82	15.35	14.40	7.26	7.02	14.25	12.44

^a^The first observation point refers to the date each participant is for the first time included in the dataset.

^b^Presence of chronic diseases includes illness reported at or before the first observation point.

Partial LE between ages 50 to 75 for men was 23.7 years in ELSA, 24.1 years in FPS, 24.5 years in GAZEL and 25.2 years in SLOSH. The corresponding figures for women were 24.5 years for ELSA, 25.0 years for FPS, 25.0 years for GAZEL and 25.5 years for SLOSH. Based on the most recent national records, the total LE at age 50 for men in England was 31.3, in Finland 30.3, in France 30.8 and in Sweden 32.0 years. Corresponding figures for women were 34.4 for England, 35.0 for Finland, 36.2 for France and 35.1 for Sweden. Thus, the differences in country-level differences in total LE at age 50 were consistent with the cohort-specific partial LEs that we observed.

**Figure 1.**
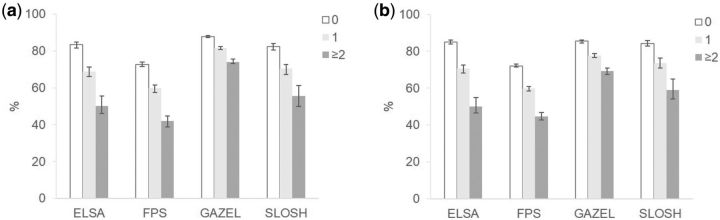
Proportion of life spent in good health between the ages of 50 and 75 by co-occurrence of behaviour-related risk factors by study cohort. a) Men, b) Women.

Table 2 shows estimates of partial LE between 50 and 75, divided into healthy and unhealthy LE based on self-reported health and by behaviour-related risk factors. There was a gradient towards shorter LE and healthy LE with increasing behaviour-related risk factors across cohorts. The differences in co-occurrence of behaviour-related risk factors were more marked for healthy LE than for partial LE in all cohorts and both sexes. In SLOSH, LE was little affected by co-occurring behaviour-related risk factors. The largest differences were observed for ELSA participants. For example, men in the ELSA cohort with no behaviour-related risk factors could expect to live 24.4 years and spend 83% of their life from 50 to 75 in good health, whereas for men with two or more behaviour-related risk factors the corresponding figures were 21.4 years and 50% in good health. In FPS and SLOSH, the differences in proportions of healthy life between co-occurring risk factor groups were also large, but in GAZEL the differences were smaller (Figure 1).

**Table 2.** Partial life expectancy, healthy life expectancy and unhealthy life expectancy based on self-reported health between the ages of 50 and 75 by co-occurrence of behaviour-related risk factors in each study cohort

	Life expectancy	95% CI		Healthy life expectancy	95% CI		Unhealthy life expectancy	95% CI		%^a^	95% CI	
Men												
ELSA												
Number of risk factors												
0	24.37	24.21	24.54	20.31	19.86	20.76	4.06	3.69	4.48	83.4	81.6	84.8
1	23.07	22.82	23.37	15.88	15.23	16.58	7.19	6.63	7.8	68.8	66.2	71.4
≥2	21.35	20.69	21.92	10.71	9.7	12.01	10.64	9.61	11.49	50.2	46.2	55.6
FPS												
Number of risk factors												
0	24.77	24.64	24.95	18.01	17.69	18.36	6.76	6.44	7.07	72.7	71.5	74
1	23.78	23.46	24.03	14.21	13.6	14.61	9.57	9.12	10.14	59.8	57.4	61.4
≥2	22.28	22.04	23.06	9.38	8.74	10.19	12.9	12.45	13.91	42.1	38.8	44.7
GAZEL												
Number of risk factors												
0	24.98	24.87	25.07	21.96	21.79	22.1	3.03	2.89	3.14	87.9	87.4	88.4
1	24.4	24.23	24.55	19.9	19.61	20.12	4.49	4.3	4.7	81.6	80.7	82.3
≥2	22.99	22.69	23.43	17.04	16.72	17.53	5.94	5.62	6.23	74.1	73.1	75.6
SLOSH												
Number of risk factors												
0	25.33	25.09	25.58	20.85	20.31	21.33	4.48	4.05	4.94	82.3	80.5	84
1	25.27	24.9	25.62	17.79	16.92	18.41	7.47	6.87	8.27	70.4	67.1	72.7
≥2	24.64	23.67	25.41	13.68	12.2	14.95	10.96	9.58	12.46	55.5	49.9	61.2
Women												
ELSA												
Number of risk factors												
0	24.99	24.91	25.16	21.22	20.96	21.57	3.77	3.48	4.06	84.9	83.8	86
1	24.29	24.07	24.45	17.14	16.52	17.66	7.15	6.68	7.67	70.6	68.3	72.4
≥2	23.01	22.65	23.41	11.52	10.64	12.79	11.49	10.56	12.38	50.1	46.4	54.8
												
FPS												
Number of risk factors												
0	25.27	25.19	25.34	18.22	18.06	18.42	7.05	6.84	7.21	72.1	71.5	73.0
1	24.64	24.52	24.79	14.7	14.42	14.99	9.94	9.65	10.27	59.7	58.4	60.8
≥2	23.95	23.6	24.25	10.67	10.18	11.25	13.28	12.72	13.78	44.5	42.6	46.9
												
GAZEL												
Number of risk factors												
0	25.34	25.13	25.41	21.64	21.27	21.79	3.7	3.55	3.95	85.4	84.4	86
1	24.88	24.66	25.05	19.3	19.01	19.66	5.58	5.26	5.83	77.6	76.5	78.8
≥2	24.01	23.56	24.36	16.67	16.1	17.2	7.34	6.91	7.81	69.4	67.4	71
SLOSH												
Number of risk factors												
0	25.54	25.38	25.73	21.54	21.18	22.01	4	3.62	4.39	84.3	82.8	85.9
1	25.54	25.24	25.75	18.78	18.11	19.43	6.76	6.02	7.43	73.5	70.8	76.3
≥2	25.01	24.07	25.53	14.79	13.48	16.19	10.22	8.67	11.41	59.1	54.2	65

^a^Proportion of life spent in good health between the ages of 50 and 75.

**Figure 2.**
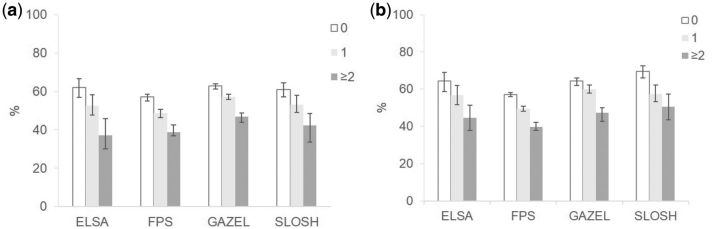
Proportion of life spent without chronic diseases between the ages of 50 and 75 by co-occurrence of behaviour-related risk factors by study cohort. a) Men, b) Women.

Results for the partial LE, chronic disease-free LE and LE with chronic diseases are shown in Table 3. Despite much higher prevalence of chronic diseases than poor self-rated health, a similar trend towards shorter chronic disease-free LE with increasing behaviour-related risk factors was observed. The differences in chronic disease-free LE between co-occurring risk factor groups were largest in ELSA. For example, men without behaviour-related risk factors in ELSA could expect to live 15 disease-free years, almost two times more than those with two or more behaviour-related risk factors. Overall, the proportions of life spent without chronic diseases were similar across cohorts and sexes (Figure 2).

**Table 3.** Partial life expectancy, chronic disease-free life expectancy and life expectancy with chronic diseases between the ages of 50 and 75 by co-occurrence of behaviour-related risk factors in each study cohort

	Life expectancy	95% CI		Chronic disease-free life expectancy	95% CI		Life expectancy with chronic diseases	95% CI		%^a^	95% CI	
Men													
ELSA													
Number of risk factors													
0	24.28	24.12	24.49	15.11	13.84	16.27	9.17	8.13	10.44		62.2	57.0	66.6
1	23.14	22.83	23.45	12.16	10.96	13.51	10.98	9.69	12.06		52.5	47.7	58.2
≥2	21.48	20.89	22.09	7.99	6.36	10.01	13.50	11.65	14.97		37.2	30	45.7
FPS													
Number of risk factors													
0	24.77	24.55	24.89	14.17	13.62	14.5	10.6	10.24	11.11		57.2	55.1	58.6
1	23.74	23.37	23.95	11.57	11.01	12.02	12.18	11.66	12.8		48.7	46.3	50.7
≥2	22.28	21.89	22.96	8.65	8.25	9.59	13.63	12.81	14.26		38.8	37	42.6
GAZEL													
Number of risk factors													
0	24.96	24.86	25.08	15.69	15.32	16.03	9.27	8.97	9.66		62.9	61.3	64.1
1	24.37	24.21	24.53	13.96	13.62	14.29	10.41	10.07	10.74		57.3	55.9	58.6
≥2	23	22.7	23.43	10.78	10.11	11.32	12.22	11.7	12.93		46.9	44	48.9
SLOSH													
Number of risk factors													
0	25.34	25.11	25.59	15.47	14.59	16.3	9.87	9.01	10.91		61.1	57.3	64.4
1	25.25	24.88	25.59	13.39	12.35	14.64	11.85	10.52	12.86		53.1	49.2	58
≥2	24.51	23.48	25.36	10.35	8.29	11.77	14.16	12.59	16.48		42.2	33.7	48.6
Women													
ELSA													
Number of risk factors													
0	24.94	24.82	25.09	16.1	14.68	17.23	8.84	7.79	10.25		64.5	58.8	69
1	24.23	24.04	24.45	13.75	12.49	15.07	10.48	9.23	11.75		56.8	51.7	62
≥2	23.16	22.74	23.52	10.36	8.73	11.88	12.81	11.15	14.43		44.7	37.7	51.3
FPS													
Number of risk factors													
0	25.24	25.17	25.32	14.4	14.13	14.67	10.85	10.57	11.13		57	55.9	58.1
1	24.61	24.49	24.78	12.19	11.84	12.5	12.42	12.11	12.81		49.5	48	50.8
≥2	23.85	23.56	24.15	9.48	9.01	10.06	14.36	13.77	14.91		39.8	37.7	42.1
GAZEL													
Number of risk factors													
0	25.28	25.16	25.44	16.28	15.65	16.72	9.00	8.62	9.62		64.4	61.9	66
1	24.89	24.69	25.09	14.93	14.39	15.5	9.96	9.41	10.49		60	57.9	62.2
≥2	23.78	23.37	24.24	11.24	10.13	12.02	12.54	11.88	13.64		47.2	42.7	50.1
SLOSH													
Number of risk factors													
0	25.52	25.34	25.69	17.71	16.82	18.57	7.82	7	8.66		69.4	66	72.6
1	25.41	25.01	25.69	14.56	13.51	15.83	10.85	9.52	11.83		57.3	53.3	62.3
≥2	24.87	24.04	25.56	12.61	10.67	14.17	12.26	10.55	14.15		50.7	43.5	57.4

^a^Proportion of life spent without chronic diseases between the ages of 50 and 75.

Healthy LE between the ages of 50 and 75 across cohorts was 20.3 and 20.7 years in men and women with no risk factors, and 12.7 and 13.4 years in those with two or more risk factors, a difference of 7.6 and 7.2 years, respectively. The corresponding differences among men and women with any single risk factor vs none were on average 3.5 years and 3.0 years for smoking, 4.7 and 4.3 years for physical inactivity and 4.2 and 4.1 years for obesity, respectively. Results regarding individual behaviour-related risk factors by cohort are shown in the online supplement (Supplementary Data, available as Supplementary Data at *IJE* online).

For men and women respectively, across cohorts, chronic disease-free LE between the ages of 50 to 75 with no risk factors was on average 15.1 years and 16.1 years, and for two or more risk factors 9.4 years and 10.9 years, a difference of 5.7 years and 5.2 years, respectively. The corresponding differences among men and women with any single risk factor vs none were for smoking on average 2.2 years and 2.3 years, for physical inactivity 2.6 and 2.2 years and for obesity 4.6 and 4.5 years, respectively (Supplementary Data, available as Supplementary Data at *IJE* online).

In multinomial logistic models, inclusion of interaction terms between sex and the combined behaviour-related risk factors did not significantly improve model fit. In general, this was also the case when interaction terms were added to the models for each individual risk factor. The only exceptions for this were: smoking in FPS and GAZEL where the increased risk of remaining unhealthy or transition to death was more marked in male smokers than female smokers; for physical inactivity and self-rated health in FPS where the increased risk of transition from good to poor health was slightly higher for inactive men than for inactive women; and for obesity and self-rated health in GAZEL where obese women were slightly less likely to recover from poor health than obese men.

Since chronic diseases were very common at the first observation point (Table 1), the analyses were repeated using modified disease outcome which divided the partial LE into LE with 0 and 1 chronic disease, and LE with two or more chronic diseases (Supplementary Data, available as Supplementary Data at *IJE* online). As expected, participants lived longer with 0–1 disease than with 0 disease, but the difference in proportions of ‘healthy life’ between two or more behaviour-related risk factors and with no risk factors was comparable between ‘years with 0–1 disease’ and ‘years without disease’.

## Discussion

This multi-cohort study showed differences in healthy and chronic disease-free LEs according to individual and co-occurring behaviour-related risk factors in men and women as well as across cohorts from England, Finland, France and Sweden. Compared with men and women with at least two of the smoking, physical inactivity and obesity risk factors, people with no risk factors could expect to live on average 8t years longer in good health and 6 years longer free of chronic diseases between the ages of 50 and 75 years. The reduction in healthy and chronic disease-free LE was greater for those with multiple behaviour-related risk factors than those with a single risk factor, a finding observed in all four cohorts.

To our knowledge this is the first study to provide healthy LE and chronic disease-free LE estimates according to multiple behaviour-related risk factors across several European countries using longitudinal data. At least two previous studies have examined the association of multiple behaviour-related risk factors with quality-adjusted life years^40^ and cognitive impairment-free life expectancy.^41^ In addition, one study examined the relation of obesity and smoking to LE and disability-free LE by using data from nine countries participating in the European Community Household Panel,^23^ although only results pooled across countries were reported. Thus, our multi-cohort study adds to the field by examining the effects of multiple behaviour-related risk factors on healthy and chronic disease-free LE in several cohorts simultaneously and showing that the findings are relatively consistent across different study populations in Europe.

The associations of multiple behaviour-related risk factors were more prominent with healthy LE and chronic disease-free LE than with LE. One explanation for this finding is ‘right censoring’, as we estimated only partial LE between ages 50 to 75. At that age, mortality is rare even among people who already developed symptoms and diseases and therefore further research is needed to examine whether differences in LE would become more pronounced with longer follow-ups.

Among the individual behaviour-related risk factors, physical inactivity was most prevalent in three of the four cohorts and was associated with the greatest reductionin healthy LE. This is a notable finding, given that we used relatively crude, dichotomized measurement due to heterogeneity in the physical activity measure between cohorts. In the ELSA and FPS, more detailed information about physical activity intensity and frequency was available, allowing us to define the cut-point for physical inactivity that was consistent with the current physical activity recommendations.^42^ Nevertheless, future studies are needed to examine the intensity and volume of physical activity in relation to healthy LE and chronic disease-free LE.

In all cohorts of this study, healthy LE was longer than chronic disease-free LE. This has also been observed in other studies using multiple types of health indicators to calculate health expectancy.^43–45^ This is expected because suboptimal self-rated health is a holistic measure and it captures a wider range of health-related phenomena beyond chronic disease.^46^ Therefore, individuals with chronic diseases may consider their health good if the disease does not hamper everyday life. A further explanation is reversibility; we allowed for recovery from suboptimal self-rated health when estimating healthy LE, but transition from the presence to the absence of chronic diseases was not allowed for when estimating chronic disease-free LE.

Direct comparison across study cohorts needs to be done cautiously. Despite careful harmonization, there was some heterogeneity in the definitions of health and chronic diseases between cohort studies, and the cohorts were also different in terms of representativeness and age.^47^ ELSA is the only study that includes a national representative sample of older individuals, whereas FPS, GAZEL and SLOSH are occupational cohorts including healthier individuals from the general population. Further sources of differences in healthy LE and chronic disease-free LE between cohorts are differences in country-specific social, economic and environmental factors that can influence health expectancy.^45^^,^^48^ In spite of these differences, the findings were relatively consistent across cohorts, suggesting that smoking, physical inactivity and obesity remain important drivers of healthy LE and chronic disease-free LE in general.

The results of the present study need to be interpreted in the context of its limitations. First, except for obesity in ELSA, all data were obtained by self-reports and are therefore subject to potential measurement errors. Self-reports may lead to under-reporting of unhealthy behaviours as well as of poor health and chronic diseases. This limitation is shared with previous studies which have also used self-reports in estimating health expectancy.7–9 Second, we were able to examine the co-occurrence of only three behaviour-related risk factors, because comparable data on alcohol consumption and diet across cohorts were not available. Further research is needed to examine whether differences in healthy LE and chronic disease-free LE would be greater when using a larger set of behaviour-related risk factors. Third, we used five chronic diseases, namely heart disease, stroke, chronic lung disease, cancer and diabetes, to estimate chronic disease-free LE. Musculoskeletal disorders, which are very common at older ages, are related to poorer functioning and quality of life and thus should be included in future studies of chronic disease-free LE. Fourth, differences in the use of health care services and care homes may contribute to differences in healthy LE and chronic disease-free LE between countries, although this is likely to be a major source of heterogeneity when estimating partial healthy LE and chronic disease-free LE from age 50 to 75. Fifth, our life expectancy analyses were conditional on reaching age of 50 and truncated at age 75. Thus, future studies are needed to investigate the association of behaviour-related risk factors with healthy LE and chronic disease-free LE starting at younger ages, and extending follow-up beyond the age of 75.

A major strength of our study is that it is based on large prospective cohort studies from four European countries, with multiple measurements of self-rated health and chronic diseases over time, long follow-up and high-quality harmonized data. We used micro-simulation to estimate healthy LE and chronic disease-free LE, which provides internally consistent results for each cohort.

In conclusion, data from four European countries show that individual and co-occurring behaviour-related risk factors are associated with reduced healthy and chronic disease-free LE. Our results support the view that reducing smoking, physical inactivity and obesity could substantially increase the time spent in good health in the population.

## Funding

This study was financially supported by the Academy of Finland (264944), the Swedish Research Council for Health for Working Life and Welfare (2007–1762 and 2009–1758) and the UK Economic and Social Research Council [ES/K01336X/1] under the ERA-AGE2 initiative. S.S. is supported by the Academy of Finland (286294 and 294154) and M.K. is supported by the MRC (K013351), NordForsk and the ESRC.

**Conflict of interest:** None declared.

## Supplementary Material

Supplementary Data

## References

[1] Kinsella K He W U.S. Census Bureau, International Population Reports, P95/09-1. An Aging World: 2008. Washington, DC: U.S. Government Printing Office, 2009.

[2] Murray CJ Barber RM Foreman KJ et al. Global, regional, and national disability-adjusted life years (DALYs) for 306 diseases and injuries and healthy life expectancy (HALE) for 188 countries, 1990–2013: quantifying the epidemiological transition. Lancet 2015;386**:** 2145–91. 26321261 10.1016/S0140-6736(15)61340-X PMC4673910

[3] Sanders BS Measuring community health levels. Am J Public Health 1964;54**:** 1063–70. 10.2105/ajph.54.7.1063 PMC1254928 14157838

[4] Wood R Sutton M Clark D McKeon A Bain M Measuring inequalities in health: the case for healthy life expectancy. J Epidemiol Community Health 2006;60**:** 1089–92. 17108308 10.1136/jech.2005.044941 PMC2465513

[5] Knoops KT de Groot LC Kromhout D et al. Mediterranean diet, lifestyle factors, and 10-year mortality in elderly European men and women: the HALE project. JAMA 2004;292**:** 1433–39. 15383513 10.1001/jama.292.12.1433

[6] Forouzanfar MH Alexander L Anderson HR et al. Global, regional, and national comparative risk assessment of 79 behavioural, environmental and occupational, and metabolic risks or clusters of risks in 188 countries, 1990–2013: a systematic analysis for the Global Burden of Disease Study 2013. Lancet 2015;386**:** 2287–323. 26364544 10.1016/S0140-6736(15)00128-2 PMC4685753

[7] Steensma C Loukine L Orpana H et al. Comparing life expectancy and health-adjusted life expectancy by body mass index category in adult Canadians: a descriptive study. Popul Health Metr 2013;11**:** 21. 24252500 10.1186/1478-7954-11-21 PMC3842774

[8] Ferrucci L Izmirlian G Leveille S et al. Smoking, physical activity, and active life expectancy. Am J Epidemiol 1999;149**:** 645–53. 10192312 10.1093/oxfordjournals.aje.a009865

[9] Tian X Tang Z Jiang J et al. Effects of smoking and smoking cessation on life expectancy in an elderly population in Beijing, China, 1992–2000: an 8-year follow-up study. J Epidemiol 2011;21**:** 376–84. 21747208 10.2188/jea.JE20110001 PMC3899437

[10] Gopinath B Flood VM Burlutsky G Mitchell P Combined influence of health behaviours on total and cause-specific mortality. Arch Intern Med 2010;170**:** 1605–07. 20876415 10.1001/archinternmed.2010.303

[11] Hamer M Bates CJ Mishra GD Multiple health behaviours and mortality risk in older adults. J Am Geriatr Soc 2011;59**:** 370–72. 21314658 10.1111/j.1532-5415.2011.03258.x PMC3398129

[12] Khaw KT Wareham N Bingham S Welch A Luben R Day N Combined impact of health behaviours and mortality in men and women: the EPIC-Norfolk prospective population study. PLoS Med 2008;5**:** e12. 18184033 10.1371/journal.pmed.0050012 PMC2174962

[13] Kvaavik E Batty GD Ursin G Huxley R Gale CR Influence of individual and combined health behaviours on total and cause-specific mortality in men and women: the United Kingdom health and lifestyle survey. Arch Intern Med 2010;170**:** 711–18. 20421558 10.1001/archinternmed.2010.76

[14] van Dam RM Li T Spiegelman D Franco OH Hu FB Combined impact of lifestyle factors on mortality: prospective cohort study in US women. BMJ 2008;337**:** a1440. 18796495 10.1136/bmj.a1440 PMC2658866

[15] Myint PK Luben RN Wareham NJ Bingham SA Khaw KT Combined effect of health behaviours and risk of first ever stroke in 20,040 men and women over 11 years’ follow-up in Norfolk cohort of European Prospective Investigation of Cancer (EPIC Norfolk): prospective population study. BMJ 2009;338**:** b349. 19228771 10.1136/bmj.b349 PMC2645849

[16] Hu FB Manson JE Stampfer MJ et al. Diet, lifestyle, and the risk of type 2 diabetes mellitus in women. N Engl J Med 2001;345**:** 790–97. 11556298 10.1056/NEJMoa010492

[17] Platz EA Willett WC Colditz GA Rimm EB Spiegelman D Giovannucci E Proportion of colon cancer risk that might be preventable in a cohort of middle-aged US men. Cancer Causes Control 2000;11**:** 579–88. 10977102 10.1023/a:1008999232442

[18] Sabia S Nabi H Kivimaki M Shipley MJ Marmot MG Singh-Manoux A Health behaviours from early to late midlife as predictors of cognitive function: The Whitehall II study. Am J Epidemiol 2009;170**:** 428–37. 19574344 10.1093/aje/kwp161 PMC2727179

[19] Sv Sabia Singh-Manoux A Hagger-Johnson G Cambois E Brunner EJ Kivimäki M Influence of individual and combined healthy behaviours on successful aging. CMAJ 2012;184**:** 1985–92. 23091184 10.1503/cmaj.121080 PMC3519184

[20] Reuser M Bonneux LG Willekens FJ Smoking kills, obesity disables: a multistate approach of the US Health and Retirement Survey. Obesity (Silver Spring) 2009;17**:** 783–89. 19165165 10.1038/oby.2008.640

[21] van Baal PH Hoogenveen RT de Wit GA Boshuizen HC Estimating health-adjusted life expectancy conditional on risk factors: results for smoking and obesity. Popul Health Metr 2006;4**:** 14. 17083719 10.1186/1478-7954-4-14 PMC1636666

[22] Stewart ST Cutler DM Rosen AB Forecasting the effects of obesity and smoking on U.S. life expectancy. N Engl J Med 2009;361**:** 2252–60. 19955525 10.1056/NEJMsa0900459 PMC4394736

[23] Majer IM Nusselder WJ Mackenbach JP Kunst AE Life expectancy and life expectancy with disability of normal weight, overweight, and obese smokers and nonsmokers in Europe. Obesity (Silver Spring) 2011;19**:** 1451–59. 21415846 10.1038/oby.2011.46

[24] World Health Organization. Global Recommendations on Physical Activity for Health. Geneva: WHO Press, 2010. 26180873

[25] Warburton DE Charlesworth S Ivey A Nettlefold L Bredin SS A systematic review of the evidence for Canada's Physical Activity Guidelines for Adults. Int J Behav Nutr Phys Act 2010;7**:** 39. 20459783 10.1186/1479-5868-7-39 PMC3583166

[26] U.S. Department of Health and Human Services. 2008 Physical Activity Guidelines for Americans. Washington, DC: U.S. Department of Health and Human Services, 2008.

[27] Warburton DE Nicol CW Bredin SS Health benefits of physical activity: the evidence. CMAJ 2006;174**:** 801–0 U.S. Department of Health and Human Services 9. 16534088 10.1503/cmaj.051351 PMC1402378

[28] Koster A Harris TB Moore SC et al. Joint associations of adiposity and physical activity with mortality: the National Institutes of Health-AARP Diet and Health Study. Am J Epidemiol 2009;169)**:** 1344–51. 19372216 10.1093/aje/kwp053 PMC2800254

[29] Koster A Patel KV Visser M et al. Joint effects of adiposity and physical activity on incident mobility limitation in older adults. J Am Geriatr Soc 2008;56**:** 636–43. 18284534 10.1111/j.1532-5415.2007.01632.x

[30] Steptoe A Breeze E Banks J Nazroo J Cohort Profile: The English Longitudinal Study of Ageing. Int J Epidemiol 2013;42**:** 1640–48. 23143611 10.1093/ije/dys168 PMC3900867

[31] Goldberg M Leclerc A Bonenfant S et al. Cohort Profile: The GAZEL Cohort Study. Int J Epidemiol 2007;36**:** 32–39. 17101614 10.1093/ije/dyl247 PMC2258334

[32] Magnusson Hanson LL Theorell T Oxenstierna G Hyde M Westerlund H Demand, control and social climate as predictors of emotional exhaustion symptoms in working Swedish men and women. Scand J Public Health 2008;36**:** 737–43. 18684778 10.1177/1403494808090164

[33] Heikkilä K Nyberg ST Fransson EI et al. Job strain and tobacco smoking: an individual-participant data meta-analysis of 166,130 adults in 15 European studies. PLoS One 2012;7**:** e35463. 22792154 10.1371/journal.pone.0035463 PMC3391192

[34] Fransson EI Heikkilä K Nyberg ST et al. Job strain as a risk factor for leisure-time physical inactivity: an individual-participant meta-analysis of up to 170,000 men and women: the IPD-Work Consortium. Am J Epidemiol 2012;176**:** 1078–89. 23144364 10.1093/aje/kws336 PMC3521479

[35] World Health Organization. Obesity: Preventing and Managing the Global Epidemic. Report of a WHO Consultation. Geneva: WHO Technical Report Series 894, 2000. 11234459

[36] Niedhammer I Chea M Psychosocial factors at work and self reported health: comparative results of cross sectional and prospective analyses of the French GAZEL cohort. Occup Environ Med 2003;60**:** 509–15. 12819285 10.1136/oem.60.7.509 PMC1740565

[37] Cai L Hayward MD Saito Y Lubitz J Hagedorn A Crimmins E Estimation of multi-state life table functions and their variability from complex survey data using the SPACE Program. Demogr Res 2010;22**:** 129–58. 20463842 10.4054/DemRes.2010.22.6 PMC2867357

[38] Cai LM Hayward MD Saito Y Lubitz J Hagedorn A Crimmins E Estimation of multi-state life table functions and their variability from complex survey data using the SPACE Program. Demogr Res 2010;22**:** 129–57. 20463842 10.4054/DemRes.2010.22.6 PMC2867357

[39] Lièvre A Brouard M Heathcote C The estimation of health expectancies from cross-longitudinal surveys. Math Popul Stud 2003;10**:** 211–48.

[40] Fransen HP May AM Beulens JW et al. Association between lifestyle factors and quality-adjusted life years in the EPIC-NL cohort. PLoS One. 2014;9**:** e111480. 25369457 10.1371/journal.pone.0111480 PMC4219750

[41] Anstey KJ Kingston A Kiely KM Luszcz MA Mitchell P Jagger C The influence of smoking, sedentary lifestyle and obesity on cognitive impairment-free life expectancy. Int J Epidemiol 2014;43**:** 1874–83. 25150976 10.1093/ije/dyu170

[42] Physical Activity Guidelines Advisory Committee. Physical Activity Guidelines Advisory Committee Report. Washington, DC: U.S. Department of Health and Human Services, 2008.

[43] Gu D Dupre ME Warner DF Zeng Y Changing health status and health expectancies among older adults in China: gender differences from 1992 to 2002. Soc Sci Med 2009;68**:** 2170–79. 19394120 10.1016/j.socscimed.2009.03.031 PMC2727476

[44] Cambois E Laborde C Romieu I Robine JM Occupational inequalities in health expectancies in France in the early 2000s: Unequal chances of reaching and living retirement in good health. Demogr Res 2011;25**:** 407–36.

[45] Jagger C Weston C Cambois E et al. Inequalities in health expectancies at older ages in the European Union: findings from the Survey of Health and Retirement in Europe (SHARE). J Epidemiol Community Health 2011;65**:** 1030–35. 21471138 10.1136/jech.2010.117705

[46] Jylhä M Guralnik JM Balfour JB Fried LP Walking difficulty, walking speed, and age as predictors of self-rated health: the Women's Health and Aging Study. J Gerontol A Biol Sci Med Sci 2001;56**:** M609–17. 11584033 10.1093/gerona/56.10.m609

[47] Pongiglione B De Stavola BL Ploubidis GB A systematic literature review of studies analyzing inequalities in health expectancy among the older population. PLoS One 2015;10**:** e0130747. 26115099 10.1371/journal.pone.0130747 PMC4482630

[48] Jagger C Gillies C Moscone F et al. Inequalities in healthy life years in the 25 countries of the European Union in 2005: a cross-national meta-regression analysis. Lancet 2008;372**:** 2124–31. 19010526 10.1016/S0140-6736(08)61594-9

