# Supplementary material for: Smoking, physical inactivity and obesity as predictors of healthy and disease-free life expectancy between ages 50 and 75: a multicohort study
Source: Int J Epidemiol. 2016 Aug 2;45(4):1260–70. doi: 10.1093/ije/dyw126 (PMC6937009; doi:10.1093/ije/dyw126)
Supplement: Supplementary Data [file dyw126_supplementary_data.zip › ije-2015-10-1457-File006.docx]

**Supplementary material**

**eTable 1. Operationalization of the behavior-related risk factors in each study cohort.**

|  | **Smoking** | **Physical inactivity** | **Obesity** |
| --- | --- | --- | --- |
| *Categorization* | *Yes: Current smoking No: Former smoking, never smoking* | *Yes: No or very little moderate or vigorous leisure-time physical activity or exercise No: Regular physical activity* | *Yes: Body mass index  ≥ 30 kg/m^2^ No: Body mass index < 30 kg/m^2^* |
| ELSA | Q: “Do you smoke cigarettes at all nowadays?”  A: “Yes”, “No” | Q: “We would like to know the type and amount of physical activity involved in your daily life. Do you take part in sports or activities that are vigorous or moderate energetic sports or activities?”  A: (1) “More than once a week”; (2) “Once a week”; (3) “One to three times a month”; (4) “Hardly ever, or never”  Subjects who responded “Hardly ever, or never” were classified as physically inactive. | Body weight and height measured by study nurse.  Body mass index was calculated as body weight divided by square of body height. |
| FPS | Q: “Do you smoke or have you previously smoked regularly, that is daily or nearly daily?” Followed by a question “Do you still smoke regularly?”  A: Subjects who responded “yes” to both questions were defined as current smokers while those who responded “no” to the latter question were defined as non-smokers. | Q: “Estimate the average weekly hours of leisure-time physical activity (including commuting) within the previous year in walking, brisk walking, jogging and running, or their equivalent activities”  A: The time spent on activity at each intensity level in hours per week was multiplied by the average energy expenditure of each activity, expressed in metabolic equivalent (MET). Physical inactivity was defined as weekly physical activity was less than 14 MET hours per week. | Q: “How tall are you?” / “How much do you weigh?”  Body mass index was calculated as body weight divided by square of body height. |
| GAZEL | Q: “Do you currently smoke?”  A: (1) “Yes, at least one cigarette per day”, (2) “Non-smoker or occasional smoker” | Q: “What distance do you currently cover on foot?”  A: (1) “Less than 500 m per week”; (2)” Between 500 m and 5 km per week” ; (3) “Between 5 km and 10 km per week”; (4) “Between 10 km and 20 km per week”; (5) “More than 20 km per week”  Subject who responded “Less than 500 m per week” or “Between 500 m and 5 km per week” were classified as physically inactive. | Q: “What is your weight?” / “What is your height?”  Body mass index was calculated as body weight divided by square of body height. |
| SLOSH | Q: “Do you smoke?”  A: (1) “Yes, every day”, (2) “Occasionally”, (3)“No”  Yes, every day and occasionally were combined into “Current smoker”. | Q: “How much exercise do you get? Include any walking or cycling you do to work.”  A: (1) “I never exercise”; (2) “I don’t exercise very much. I take odd walks”; (3) “I exercise now and again”; (4) “I exercise regularly”  Subjects who responded “I never exercise” or “I don’t exercise very much” were classified as physically inactive. | Q: “How tall are you?” / “How much do you weigh?”  Body mass index was calculated as body weight divided by square of body height. |

**eTable 2. Partial life expectancy, healthy life expectancy and unhealthy life expectancy based on self-reported health between the ages of 50 and 75 by smoking, physical inactivity and obesity in each study cohort.**

|  | Life expectancy | 95% CI | |  | Healthy life expectancy | 95% CI | |  | Unhealthy life expectancy | 95% CI | |  | %* | 95% CI | |
| --- | --- | --- | --- | --- | --- | --- | --- | --- | --- | --- | --- | --- | --- | --- | --- |
| **MEN** |  |  |  |  |  |  |  |  |  |  |  |  |  |  |  |
|  | ELSA | | | | | | | | | | | | | | |
| Smoking |  |  |  |  |  |  |  |  |  |  |  |  |  |  |  |
| Yes | 21.86 | 21.36 | 22.27 |  | 13.95 | 13.07 | 14.73 |  | 7.92 | 7.36 | 8.6 |  | 63.8 | 60.3 | 66.6 |
| No | 24.19 | 23.98 | 24.4 |  | 19.09 | 18.65 | 19.46 |  | 5.1 | 4.81 | 5.48 |  | 78.9 | 77.3 | 80.1 |
| Physical inactivity |  |  |  |  |  |  |  |  |  |  |  |  |  |  |  |
| Yes | 21.6 | 20.96 | 22.06 |  | 11.24 | 9.65 | 12.32 |  | 10.35 | 9.41 | 11.43 |  | 52.1 | 46 | 56.4 |
| No | 23.83 | 23.63 | 24.06 |  | 18.43 | 18 | 18.85 |  | 5.4 | 5.09 | 5.73 |  | 77.3 | 75.9 | 78.7 |
| Obesity |  |  |  |  |  |  |  |  |  |  |  |  |  |  |  |
| Yes | 23.3 | 22.92 | 23.55 |  | 15.47 | 14.82 | 16.14 |  | 7.83 | 7.21 | 8.36 |  | 66.4 | 64 | 69.1 |
| No | 23.88 | 23.63 | 24.06 |  | 18.76 | 18.36 | 19.21 |  | 5.12 | 4.74 | 5.41 |  | 78.6 | 77.2 | 80.2 |
|  |  |  |  |  |  |  |  |  |  |  |  |  |  |  |  |
|  | FPS | | | | | | | | | | | | | | |
| Smoking |  |  |  |  |  |  |  |  |  |  |  |  |  |  |  |
| Yes | 22.35 | 21.86 | 22.83 |  | 12.16 | 11.73 | 13 |  | 10.19 | 9.44 | 10.57 |  | 54.4 | 52.8 | 57.8 |
| No | 24.6 | 24.42 | 24.76 |  | 16.48 | 16.08 | 16.84 |  | 8.12 | 7.78 | 8.47 |  | 67 | 65.6 | 68.3 |
| Physical inactivity |  |  |  |  |  |  |  |  |  |  |  |  |  |  |  |
| Yes | 23.41 | 23.17 | 23.86 |  | 12.06 | 11.79 | 12.93 |  | 11.35 | 10.7 | 11.76 |  | 51.5 | 50.1 | 54.5 |
| No | 24.39 | 24.14 | 24.55 |  | 16.67 | 16.28 | 17 |  | 7.73 | 7.4 | 8.05 |  | 68.3 | 67 | 69.6 |
| Obesity |  |  |  |  |  |  |  |  |  |  |  |  |  |  |  |
| Yes | 23.35 | 23.05 | 23.83 |  | 10.83 | 10.06 | 11.32 |  | 12.52 | 12.1 | 13.41 |  | 46.4 | 42.9 | 48.3 |
| No | 24.33 | 24.09 | 24.49 |  | 16.56 | 16.14 | 16.82 |  | 7.77 | 7.49 | 8.11 |  | 68.1 | 66.7 | 69.1 |
|  |  |  |  |  |  |  |  |  |  |  |  |  |  |  |  |
|  | GAZEL | | | | | | | | | | | | | | |
| Smoking |  |  |  |  |  |  |  |  |  |  |  |  |  |  |  |
| Yes | 23.42 | 23.13 | 23.7 |  | 18.92 | 18.56 | 19.21 |  | 4.5 | 4.26 | 4.74 |  | 80.8 | 79.7 | 81.8 |
| No | 24.78 | 24.68 | 24.87 |  | 20.96 | 20.77 | 21.13 |  | 3.82 | 3.7 | 3.98 |  | 84.6 | 83.9 | 85.1 |
| Physical inactivity |  |  |  |  |  |  |  |  |  |  |  |  |  |  |  |
| Yes | 24.09 | 23.94 | 24.26 |  | 18.88 | 18.59 | 19.1 |  | 5.21 | 5.03 | 5.45 |  | 78.4 | 77.4 | 79.1 |
| No | 24.79 | 24.66 | 24.88 |  | 21.62 | 21.42 | 21.74 |  | 3.17 | 3.06 | 3.32 |  | 87.2 | 86.6 | 87.7 |
| Obesity |  |  |  |  |  |  |  |  |  |  |  |  |  |  |  |
| Yes | 23.69 | 23.33 | 24.1 |  | 17.8 | 17.27 | 18.36 |  | 5.89 | 5.48 | 6.36 |  | 75.1 | 73.2 | 76.8 |
| No | 24.55 | 24.47 | 24.68 |  | 20.74 | 20.61 | 20.93 |  | 3.81 | 3.65 | 3.92 |  | 84.5 | 84.1 | 85.1 |
|  |  |  |  |  |  |  |  |  |  |  |  |  |  |  |  |
|  | SLOSH | | | | | | | | | | | | | | |
| Smoking |  |  |  |  |  |  |  |  |  |  |  |  |  |  |  |
| Yes | 24.8 | 24.14 | 25.36 |  | 17.11 | 16.17 | 18.25 |  | 7.69 | 6.61 | 8.63 |  | 69 | 65.2 | 73 |
| No | 25.36 | 25.04 | 25.53 |  | 19.53 | 19.05 | 20.01 |  | 5.83 | 5.32 | 6.2 |  | 77 | 75.5 | 79 |
| Physical inactivity |  |  |  |  |  |  |  |  |  |  |  |  |  |  |  |
| Yes | 24.9 | 24.42 | 25.51 |  | 15.61 | 14.78 | 16.74 |  | 9.29 | 8.17 | 10.18 |  | 62.7 | 59.5 | 67.3 |
| No | 25.29 | 25.07 | 25.55 |  | 20.15 | 19.64 | 20.55 |  | 5.14 | 4.81 | 5.63 |  | 79.7 | 77.7 | 80.9 |
| Obesity |  |  |  |  |  |  |  |  |  |  |  |  |  |  |  |
| Yes | 25.38 | 24.85 | 25.84 |  | 15.17 | 13.91 | 16.1 |  | 10.21 | 9.32 | 11.53 |  | 59.8 | 54.8 | 63.1 |
| No | 25.21 | 25.01 | 25.43 |  | 19.87 | 19.4 | 20.34 |  | 5.34 | 4.95 | 5.83 |  | 78.8 | 77 | 80.4 |
|  |  |  |  |  |  |  |  |  |  |  |  |  |  |  |  |
| **WOMEN** |  |  |  |  |  |  |  |  |  |  |  |  |  |  |  |
|  | ELSA | | | | | | | | | | | | | | |
| Smoking |  |  |  |  |  |  |  |  |  |  |  |  |  |  |  |
| Yes | 23.25 | 22.93 | 23.57 |  | 14.79 | 13.94 | 15.43 |  | 8.47 | 7.97 | 9.13 |  | 63.6 | 60.4 | 65.9 |
| No | 24.85 | 24.71 | 24.96 |  | 19.66 | 19.26 | 20.01 |  | 5.19 | 4.86 | 5.54 |  | 79.1 | 77.7 | 80.5 |
| Physical inactivity |  |  |  |  |  |  |  |  |  |  |  |  |  |  |  |
| Yes | 23.22 | 22.93 | 23.6 |  | 12.61 | 11.58 | 13.76 |  | 10.61 | 9.73 | 11.53 |  | 54.3 | 50 | 58.6 |
| No | 24.68 | 24.53 | 24.82 |  | 19.35 | 18.91 | 19.76 |  | 5.33 | 4.99 | 5.68 |  | 78.4 | 76.9 | 79.8 |
| Obesity |  |  |  |  |  |  |  |  |  |  |  |  |  |  |  |
| Yes | 24.16 | 23.98 | 24.39 |  | 15.77 | 15.27 | 16.56 |  | 8.39 | 7.73 | 8.83 |  | 65.3 | 63.4 | 68.1 |
| No | 24.63 | 24.48 | 24.77 |  | 19.55 | 19.14 | 19.96 |  | 5.08 | 4.73 | 5.44 |  | 79.4 | 77.9 | 80.8 |
|  |  |  |  |  |  |  |  |  |  |  |  |  |  |  |  |
|  | FPS | | | | | | | | | | | | | | |
| Smoking |  |  |  |  |  |  |  |  |  |  |  |  |  |  |  |
| Yes | 23.72 | 23.45 | 23.95 |  | 13.64 | 13.15 | 14.01 |  | 10.08 | 9.73 | 10.5 |  | 57.5 | 55.7 | 59 |
| No | 25.14 | 25.06 | 25.2 |  | 16.93 | 16.79 | 17.15 |  | 8.21 | 7.99 | 8.35 |  | 67.3 | 66.8 | 68.2 |
| Physical inactivity |  |  |  |  |  |  |  |  |  |  |  |  |  |  |  |
| Yes | 24.64 | 24.46 | 24.8 |  | 13.67 | 13.31 | 14.01 |  | 10.97 | 10.62 | 11.34 |  | 55.5 | 54.1 | 56.8 |
| No | 25.07 | 24.98 | 25.14 |  | 17.23 | 17.07 | 17.43 |  | 7.84 | 7.63 | 7.98 |  | 68.7 | 68.1 | 69.5 |
| Obesity |  |  |  |  |  |  |  |  |  |  |  |  |  |  |  |
| Yes | 24.64 | 24.37 | 24.79 |  | 11.71 | 11.23 | 12.08 |  | 12.93 | 12.5 | 13.36 |  | 47.5 | 45.7 | 49.1 |
| No | 25.03 | 24.96 | 25.11 |  | 17.26 | 17.1 | 17.48 |  | 7.77 | 7.59 | 7.93 |  | 68.9 | 68.3 | 69.7 |
|  |  |  |  |  |  |  |  |  |  |  |  |  |  |  |  |
|  | GAZEL | | | | | | | | | | | | | | |
| Smoking |  |  |  |  |  |  |  |  |  |  |  |  |  |  |  |
| Yes | 24.16 | 23.69 | 24.48 |  | 18.49 | 17.92 | 18.95 |  | 5.67 | 5.3 | 6.08 |  | 76.5 | 74.7 | 77.9 |
| No | 25.05 | 24.91 | 25.26 |  | 20.31 | 20.03 | 20.6 |  | 4.75 | 4.53 | 5.02 |  | 81 | 80 | 82 |
| Physical inactivity |  |  |  |  |  |  |  |  |  |  |  |  |  |  |  |
| Yes | 24.74 | 24.46 | 24.92 |  | 18.5 | 18.14 | 18.81 |  | 6.24 | 5.93 | 6.51 |  | 74.8 | 73.7 | 76 |
| No | 25.16 | 25.03 | 25.32 |  | 21.32 | 21.09 | 21.61 |  | 3.84 | 3.62 | 4.04 |  | 84.7 | 83.9 | 85.6 |
|  |  |  |  |  |  |  |  |  |  |  |  |  |  |  |  |
| Obesity |  |  |  |  |  |  |  |  |  |  |  |  |  |  |  |
| Yes | 24.65 | 24.01 | 24.81 |  | 17.27 | 16.45 | 17.8 |  | 7.38 | 6.79 | 7.82 |  | 70.1 | 67.8 | 72.2 |
| No | 24.99 | 24.84 | 25.14 |  | 20.3 | 19.97 | 20.5 |  | 4.7 | 4.54 | 4.98 |  | 81.2 | 80.1 | 81.9 |
|  |  |  |  |  |  |  |  |  |  |  |  |  |  |  |  |
|  | SLOSH | | | | | | | | | | | | | | |
| Smoking |  |  |  |  |  |  |  |  |  |  |  |  |  |  |  |
| Yes | 25.12 | 24.72 | 25.56 |  | 18.55 | 17.67 | 19.42 |  | 6.57 | 5.86 | 7.45 |  | 73.9 | 70.4 | 76.7 |
| No | 25.54 | 25.34 | 25.72 |  | 20.72 | 20.26 | 21.15 |  | 4.83 | 4.38 | 5.25 |  | 81.1 | 79.4 | 82.8 |
| Physical inactivity |  |  |  |  |  |  |  |  |  |  |  |  |  |  |  |
| Yes | 25.26 | 24.84 | 25.67 |  | 16.64 | 15.64 | 17.57 |  | 8.62 | 7.69 | 9.65 |  | 65.9 | 62.1 | 69.6 |
| No | 25.52 | 25.31 | 25.71 |  | 20.86 | 20.44 | 21.22 |  | 4.66 | 4.35 | 5.06 |  | 81.8 | 80.3 | 83 |
| Obesity |  |  |  |  |  |  |  |  |  |  |  |  |  |  |  |
| Yes | 25.59 | 25.2 | 25.86 |  | 16.73 | 15.66 | 17.58 |  | 8.86 | 8.08 | 10.08 |  | 65.4 | 60.9 | 68.3 |
| No | 25.46 | 25.27 | 25.69 |  | 20.79 | 20.38 | 21.26 |  | 4.67 | 4.25 | 5.03 |  | 81.6 | 80.2 | 83.3 |
|  |  |  |  |  |  |  |  |  |  |  |  |  |  |  |  |

*Notes*: * Proportion of life spent in good health between the ages of 50 and 75.

**eTable 3. Partial life expectancy, chronic disease-free life expectancy and life expectancy with chronic diseases between the ages of 50 and 75 by smoking, physical inactivity and obesity in each study cohort.**

|  | Life expectancy | 95% CI | |  | Chronic disease-free life expectancy | 95% CI | |  | Life expectancy with chronic diseases | 95% CI | |  | %* | 95% CI | |
| --- | --- | --- | --- | --- | --- | --- | --- | --- | --- | --- | --- | --- | --- | --- | --- |
| **MEN** |  |  |  |  |  |  |  |  |  |  |  |  |  |  |  |
|  | ELSA | | | | | | | | | | | | | | |
| Smoking |  |  |  |  |  |  |  |  |  |  |  |  |  |  |  |
| Yes | 21.91 | 21.51 | 22.18 |  | 10.74 | 9.94 | 11.65 |  | 11.17 | 10 | 12.08 |  | 49 | 45.2 | 53.8 |
| No | 24.16 | 23.95 | 24.26 |  | 14.28 | 12.9 | 15.02 |  | 9.87 | 9.11 | 11.11 |  | 59.1 | 53.7 | 62.2 |
| Physical inactivity |  |  |  |  |  |  |  |  |  |  |  |  |  |  |  |
| Yes | 21.69 | 21.66 | 22.24 |  | 9.12 | 7.98 | 10.34 |  | 12.57 | 11.68 | 14.16 |  | 42 | 35.9 | 46.6 |
| No | 23.77 | 23.64 | 24.03 |  | 13.74 | 13.25 | 14.59 |  | 10.03 | 9.38 | 10.58 |  | 57.8 | 55.6 | 60.8 |
| Obesity |  |  |  |  |  |  |  |  |  |  |  |  |  |  |  |
| Yes | 23.3 | 23.15 | 23.88 |  | 11.45 | 10.68 | 12.89 |  | 11.84 | 10.87 | 12.73 |  | 49.2 | 45.7 | 54 |
| No | 23.83 | 23.68 | 24.05 |  | 14.22 | 13.19 | 15.46 |  | 9.61 | 8.47 | 10.65 |  | 59.7 | 55.3 | 64.6 |
|  |  |  |  |  |  |  |  |  |  |  |  |  |  |  |  |
|  | FPS | | | | | | | | | | | | | | |
| Smoking |  |  |  |  |  |  |  |  |  |  |  |  |  |  |  |
| Yes | 22.17 | 21.8 | 22.49 |  | 11.17 | 10.72 | 11.69 |  | 11 | 10.6 | 11.54 |  | 50.4 | 48.2 | 52.3 |
| No | 24.56 | 24.4 | 24.71 |  | 12.9 | 12.62 | 13.26 |  | 11.66 | 11.31 | 11.97 |  | 52.5 | 51.4 | 53.8 |
| Physical inactivity |  |  |  |  |  |  |  |  |  |  |  |  |  |  |  |
| Yes | 23.34 | 23.14 | 23.76 |  | 10.91 | 10.23 | 11.47 |  | 12.43 | 11.79 | 13.11 |  | 46.7 | 44 | 49.3 |
| No | 24.22 | 24.1 | 24.42 |  | 13.02 | 12.71 | 13.38 |  | 11.19 | 10.88 | 11.65 |  | 53.8 | 52.3 | 55 |
| Obesity |  |  |  |  |  |  |  |  |  |  |  |  |  |  |  |
| Yes | 23.51 | 22.84 | 23.75 |  | 8.23 | 7.62 | 8.68 |  | 15.28 | 14.37 | 15.96 |  | 35 | 32.9 | 37.6 |
| No | 24.2 | 23.93 | 24.33 |  | 13.29 | 13.03 | 13.72 |  | 10.91 | 10.37 | 11.24 |  | 54.9 | 53.7 | 57 |
|  |  |  |  |  |  |  |  |  |  |  |  |  |  |  |  |
|  | GAZEL | | | | | | | | | | | | | | |
| Smoking |  |  |  |  |  |  |  |  |  |  |  |  |  |  |  |
| Yes | 23.48 | 23.2 | 23.59 |  | 12.6 | 12.18 | 13.02 |  | 10.88 | 10.21 | 11.17 |  | 53.7 | 52.1 | 56 |
| No | 24.74 | 24.65 | 24.79 |  | 14.84 | 14.67 | 15.17 |  | 9.9 | 9.6 | 10.1 |  | 60 | 59.2 | 61.2 |
| Physical inactivity |  |  |  |  |  |  |  |  |  |  |  |  |  |  |  |
| Yes | 24.1 | 23.85 | 24.16 |  | 13 | 12.68 | 13.48 |  | 11.1 | 10.61 | 11.36 |  | 53.9 | 52.9 | 56 |
| No | 24.78 | 24.66 | 24.84 |  | 15.22 | 14.82 | 15.47 |  | 9.55 | 9.34 | 9.85 |  | 61.4 | 60.1 | 62.3 |
| Obesity |  |  |  |  |  |  |  |  |  |  |  |  |  |  |  |
| Yes | 23.69 | 23.46 | 24.1 |  | 10.3 | 9.67 | 10.93 |  | 13.39 | 12.88 | 14.29 |  | 43.5 | 40.5 | 45.8 |
| No | 24.55 | 24.48 | 24.64 |  | 14.7 | 14.55 | 14.96 |  | 9.85 | 9.62 | 10.08 |  | 59.9 | 59.1 | 60.9 |
|  |  |  |  |  |  |  |  |  |  |  |  |  |  |  |  |
|  | SLOSH | | | | | | | | | | | | | | |
| Smoking |  |  |  |  |  |  |  |  |  |  |  |  |  |  |  |
| Yes | 24.66 | 24.08 | 24.98 |  | 13.17 | 11.26 | 14.1 |  | 11.49 | 10.61 | 13.49 |  | 53.4 | 45.5 | 57.1 |
| No | 25.37 | 25.19 | 25.53 |  | 14.47 | 13.49 | 14.87 |  | 10.9 | 10.53 | 11.84 |  | 57 | 53.6 | 58.5 |
| Physical inactivity |  |  |  |  |  |  |  |  |  |  |  |  |  |  |  |
| Yes | 24.96 | 24.62 | 25.67 |  | 13 | 11.69 | 14.41 |  | 11.95 | 10.98 | 13.21 |  | 52.1 | 46.9 | 56.7 |
| No | 25.35 | 25.06 | 25.46 |  | 14.6 | 13.95 | 15.43 |  | 10.75 | 9.92 | 11.27 |  | 57.6 | 55.3 | 60.9 |
| Obesity |  |  |  |  |  |  |  |  |  |  |  |  |  |  |  |
| Yes | 25.46 | 24.84 | 25.81 |  | 9.07 | 7.65 | 10.03 |  | 16.4 | 15.54 | 18.09 |  | 35.6 | 29.7 | 38.9 |
| No | 25.22 | 24.91 | 25.31 |  | 15.17 | 14.42 | 15.88 |  | 10.05 | 9.24 | 10.67 |  | 60.2 | 57.5 | 63.2 |
|  |  |  |  |  |  |  |  |  |  |  |  |  |  |  |  |
| **WOMEN** |  |  |  |  |  |  |  |  |  |  |  |  |  |  |  |
|  | ELSA | | | | | | | | | | | | | | |
| Smoking |  |  |  |  |  |  |  |  |  |  |  |  |  |  |  |
| Yes | 23.3 | 22.96 | 23.5 |  | 12.66 | 11.72 | 13.94 |  | 10.65 | 9.52 | 11.37 |  | 54.3 | 50.7 | 59.3 |
| No | 24.76 | 24.67 | 24.87 |  | 15.17 | 14.01 | 16.48 |  | 9.59 | 8.38 | 10.69 |  | 61.3 | 56.7 | 66.3 |
| Physical inactivity |  |  |  |  |  |  |  |  |  |  |  |  |  |  |  |
| Yes | 23.42 | 23.21 | 23.85 |  | 11.71 | 10.6 | 13.63 |  | 11.72 | 10.06 | 12.91 |  | 50 | 45.3 | 57.5 |
| No | 24.59 | 24.5 | 24.77 |  | 14.89 | 13.8 | 16.03 |  | 9.7 | 8.68 | 10.83 |  | 60.5 | 56 | 64.9 |
| Obesity |  |  |  |  |  |  |  |  |  |  |  |  |  |  |  |
| Yes | 24.54 | 24.33 | 24.66 |  | 9.5 | 9.11 | 9.8 |  | 15.03 | 14.72 | 15.5 |  | 38.7 | 37.1 | 40 |
| No | 24.99 | 24.91 | 25.04 |  | 13.91 | 13.72 | 14.05 |  | 11.08 | 10.97 | 11.3 |  | 55.6 | 54.9 | 56.1 |
|  |  |  |  |  |  |  |  |  |  |  |  |  |  |  |  |
|  | FPS | | | | | | | | | | | | | | |
| Smoking |  |  |  |  |  |  |  |  |  |  |  |  |  |  |  |
| Yes | 23.66 | 23.32 | 23.82 |  | 11.57 | 11.05 | 12.16 |  | 12.09 | 11.45 | 12.58 |  | 48.9 | 46.8 | 51.5 |
| No | 25.08 | 25.03 | 25.2 |  | 13.51 | 13.37 | 13.82 |  | 11.57 | 11.29 | 11.77 |  | 53.9 | 53.2 | 55 |
| Physical inactivity |  |  |  |  |  |  |  |  |  |  |  |  |  |  |  |
| Yes | 24.59 | 24.42 | 24.71 |  | 11.79 | 11.34 | 12.06 |  | 12.8 | 12.52 | 13.24 |  | 47.9 | 46.2 | 49 |
| No | 25.04 | 24.94 | 25.1 |  | 13.66 | 13.49 | 13.85 |  | 11.38 | 11.17 | 11.56 |  | 54.5 | 53.9 | 55.3 |
| Obesity |  |  |  |  |  |  |  |  |  |  |  |  |  |  |  |
| Yes | 24.65 | 24.43 | 24.84 |  | 4.01 | 3.78 | 4.33 |  | 20.64 | 20.28 | 20.95 |  | 16.3 | 15.4 | 17.6 |
| No | 25.03 | 24.99 | 25.12 |  | 7.67 | 7.45 | 7.82 |  | 17.36 | 17.23 | 17.62 |  | 30.6 | 29.8 | 31.2 |
|  |  |  |  |  |  |  |  |  |  |  |  |  |  |  |  |
|  | GAZEL | | | | | | | | | | | | | | |
| Smoking |  |  |  |  |  |  |  |  |  |  |  |  |  |  |  |
| Yes | 24.1 | 23.76 | 24.31 |  | 12.39 | 11.24 | 12.68 |  | 11.71 | 11.27 | 12.97 |  | 51.4 | 46.6 | 52.9 |
| No | 25.13 | 25 | 25.2 |  | 15.42 | 15.25 | 16 |  | 9.7 | 9.07 | 9.82 |  | 61.4 | 60.8 | 63.8 |
| Physical inactivity |  |  |  |  |  |  |  |  |  |  |  |  |  |  |  |
| Yes | 24.7 | 24.63 | 24.84 |  | 14.23 | 13.92 | 14.74 |  | 10.47 | 9.91 | 10.79 |  | 57.6 | 56.3 | 59.8 |
| No | 25.18 | 25.11 | 25.26 |  | 15.69 | 15.24 | 16.01 |  | 9.49 | 9.17 | 9.98 |  | 62.3 | 60.5 | 63.6 |
| Obesity |  |  |  |  |  |  |  |  |  |  |  |  |  |  |  |
| Yes | 24.38 | 23.95 | 24.61 |  | 11.08 | 10.36 | 12.07 |  | 13.29 | 12.49 | 13.84 |  | 45.5 | 42.7 | 49.1 |
| No | 25.03 | 24.9 | 25.13 |  | 15.37 | 14.88 | 15.59 |  | 9.66 | 9.41 | 10.13 |  | 61.4 | 59.5 | 62.3 |
|  |  |  |  |  |  |  |  |  |  |  |  |  |  |  |  |
|  | SLOSH | | | | | | | | | | | | | | |
| Smoking |  |  |  |  |  |  |  |  |  |  |  |  |  |  |  |
| Yes | 24.92 | 24.07 | 25.34 |  | 15.04 | 13.5 | 15.42 |  | 9.88 | 9.21 | 10.73 |  | 60.3 | 56.1 | 62 |
| No | 25.53 | 25.31 | 25.71 |  | 16.73 | 16.22 | 17 |  | 8.8 | 8.44 | 9.35 |  | 65.5 | 63.4 | 66.8 |
| Physical inactivity |  |  |  |  |  |  |  |  |  |  |  |  |  |  |  |
| Yes | 25.25 | 24.93 | 25.78 |  | 14.49 | 13.62 | 16.21 |  | 10.76 | 9.18 | 11.41 |  | 57.4 | 54.4 | 63.8 |
| No | 25.49 | 25.15 | 25.54 |  | 16.72 | 16 | 17.35 |  | 8.77 | 8.17 | 9.44 |  | 65.6 | 62.9 | 68 |
| Obesity |  |  |  |  |  |  |  |  |  |  |  |  |  |  |  |
| Yes | 25.59 | 25.17 | 25.91 |  | 11.37 | 9.92 | 12.55 |  | 14.22 | 13.13 | 15.62 |  | 44.4 | 39.4 | 48.9 |
| No | 25.41 | 25.19 | 25.7 |  | 17.17 | 16.47 | 18.42 |  | 8.24 | 7.27 | 8.86 |  | 67.6 | 65 | 71.7 |
|  |  |  |  |  |  |  |  |  |  |  |  |  |  |  |  |

*Notes*: * Proportion of life spent without chronic health conditions between the ages of 50 and 75.

**eTable 4. Partial life expectancy, life expectancy with 0 or 1 chronic disease and life expectancy with 2 or more chronic disease between the ages of 50 and 75 by co-occurrence of behavior-related risk factors in each study cohort.**

|  | Life expectancy | 95% CI | |  | Life expectancy with 0-1 chronic disease | 95% CI | |  | Life expectancy with 2 or more chronic diseases | | 95% CI | | |  | %* | | 95% CI | | |  |
| --- | --- | --- | --- | --- | --- | --- | --- | --- | --- | --- | --- | --- | --- | --- | --- | --- | --- | --- | --- | --- |
| **Men** |  |  |  |  |  |  |  |  |  | |  |  | |  |  | |  | |  |  |
| ELSA |  |  |  |  |  |  |  |  |  | |  |  | |  |  | |  | |  |  |
| Number of risk factors |  |  |  |  |  |  |  |  |  | |  |  | |  |  | |  | |  |  |
| 0 | 24.36 | 24.23 | 24.46 |  | 22.86 | 22.29 | 23.3 |  | 1.49 | | 1.08 | 1.97 | |  | 93.9 | | 91.9 | | 95.6 |  |
| 1 | 23.36 | 22.82 | 23.57 |  | 21.4 | 20.74 | 21.94 |  | 1.95 | | 1.54 | 2.24 | |  | 91.6 | | 90.2 | | 93.4 |  |
| ≥2 | 21.76 | 21.29 | 22.33 |  | 17.21 | 15.5 | 20.55 |  | 4.55 | | 1.78 | 6.11 | |  | 79.1 | | 72.1 | | 92 |  |
|  |  |  |  |  |  |  |  |  |  | |  |  | |  |  | |  | |  |  |
| FPS |  |  |  |  |  |  |  |  |  | |  |  | |  |  | |  | |  |  |
| Number of risk factors |  |  |  |  |  |  |  |  |  | |  |  | |  |  | |  | |  |  |
| 0 | 24.75 | 24.57 | 24.87 |  | 21.78 | 21.48 | 22.02 |  | 2.97 | | 2.74 | 3.25 | |  | 88 | | 86.9 | | 88.9 |  |
| 1 | 23.79 | 23.46 | 23.92 |  | 19.51 | 19.14 | 19.94 |  | 4.28 | | 3.83 | 4.53 | |  | 82 | | 80.9 | | 83.7 |  |
| ≥2 | 22.29 | 22.04 | 23.12 |  | 16.8 | 16.32 | 17.55 |  | 5.49 | | 4.99 | 6.34 | |  | 75.4 | | 72.2 | | 77.6 |  |
|  |  |  |  |  |  |  |  |  |  | |  |  | |  |  | |  | |  |  |
| GAZEL |  |  |  |  |  |  |  |  |  | |  |  | |  |  | |  | |  |  |
| Number of risk factors |  |  |  |  |  |  |  |  |  | |  |  | |  |  | |  | |  |  |
| 0 | 24.97 | 24.83 | 25.09 |  | 23.86 | 23.72 | 24.06 |  | 1.11 | | 0.97 | 1.21 | |  | 95.6 | | 95.1 | | 96.1 |  |
| 1 | 24.35 | 24.15 | 24.56 |  | 23 | 22.77 | 23.21 |  | 1.36 | | 1.27 | 1.52 | |  | 94.4 | | 93.8 | | 94.8 |  |
| ≥2 | 23.1 | 22.79 | 23.44 |  | 20.55 | 20.14 | 20.8 |  | 2.55 | | 2.29 | 2.85 | |  | 89 | | 87.7 | | 90 |  |
|  |  |  |  |  |  |  |  |  |  | |  |  | |  |  | |  | |  |  |
| SLOSH^†^ |  |  |  |  |  |  |  |  |  | |  |  | |  |  | |  | |  |  |
| Number of risk factors |  |  |  |  |  |  |  |  |  | |  |  | |  |  | |  | |  |  |
| 0 | 25.38 | 25 | 25.56 |  | 24.08 | 23.64 | 24.26 |  | 1.3 | | 1.14 | 1.69 | |  | 94.9 | | 93.3 | | 95.4 |  |
| 1 | 25.27 | 25.01 | 25.51 |  | 22.29 | 21.54 | 22.86 |  | 2.98 | | 2.62 | 3.7 | |  | 88.2 | | 85.4 | | 89.7 |  |
| ≥2 | 24.41 | 23.77 | 25.9 |  | 19.9 | 17.98 | 21.62 |  | 4.51 | | 3.39 | 6.15 | |  | 81.5 | | 74.5 | | 86 |  |
|  |  |  |  |  |  |  |  |  |  | |  |  | |  |  | |  | |  |  |
| **Women** |  |  |  |  |  |  |  |  | |  |  | |  | |  |  | |  |  | |
| ELSA |  |  |  |  |  |  |  |  | |  |  | |  | |  |  | |  |  | |
| Number of risk factors |  |  |  |  |  |  |  |  | |  |  | |  | |  |  | |  |  | |
| 0 | 25 | 24.89 | 25.12 |  | 24.38 | 24.11 | 24.77 |  | | 0.62 | 0.33 | | 0.99 | |  | 97.5 | | 96 | 98.7 | |
| 1 | 24.3 | 24.12 | 24.46 |  | 22.86 | 22.35 | 23.41 |  | | 1.45 | 0.98 | | 1.86 | |  | 94 | | 92.4 | 96 | |
| ≥2 | 23.33 | 22.93 | 23.66 |  | 20.34 | 18.54 | 22.21 |  | | 2.99 | 1.21 | | 4.62 | |  | 87.2 | | 80 | 94.8 | |
|  |  |  |  |  |  |  |  |  | |  |  | |  | |  |  | |  |  | |
| FPS |  |  |  |  |  |  |  |  | |  |  | |  | |  |  | |  |  | |
| Number of risk factors |  |  |  |  |  |  |  |  | |  |  | |  | |  |  | |  |  | |
| 0 | 25.26 | 25.19 | 25.35 |  | 22.05 | 21.69 | 22.17 |  | | 3.21 | 3.07 | | 3.51 | |  | 87.3 | | 86.1 | 87.8 | |
| 1 | 24.66 | 24.52 | 24.78 |  | 20.2 | 19.91 | 20.51 |  | | 4.47 | 4.06 | | 4.72 | |  | 81.9 | | 80.9 | 83.5 | |
| ≥2 | 23.94 | 23.66 | 24.31 |  | 18.08 | 17.62 | 18.38 |  | | 5.87 | 5.54 | | 6.32 | |  | 75.5 | | 73.7 | 76.6 | |
|  |  |  |  |  |  |  |  |  | |  |  | |  | |  |  | |  |  | |
| GAZEL |  |  |  |  |  |  |  |  | |  |  | |  | |  |  | |  |  | |
| Number of risk factors |  |  |  |  |  |  |  |  | |  |  | |  | |  |  | |  |  | |
| 0 | 25.27 | 25.18 | 25.44 |  | 24.29 | 24.13 | 24.59 |  | | 0.97 | 0.71 | | 1.17 | |  | 96.1 | | 95.4 | 97.2 | |
| 1 | 24.86 | 24.7 | 25.07 |  | 23.69 | 23.52 | 23.95 |  | | 1.17 | 0.97 | | 1.43 | |  | 95.3 | | 94.3 | 96.1 | |
| ≥2 | 23.73 | 23.54 | 24.25 |  | 21.44 | 21.17 | 22.11 |  | | 2.29 | 1.85 | | 2.65 | |  | 90.3 | | 88.8 | 92.2 | |
|  |  |  |  |  |  |  |  |  | |  |  | |  | |  |  | |  |  | |
| SLOSH^†^ |  |  |  |  |  |  |  |  | |  |  | |  | |  |  | |  |  | |
| Number of risk factors |  |  |  |  |  |  |  |  | |  |  | |  | |  |  | |  |  | |
| 0 | 25.56 | 25.22 | 25.7 |  | 24.52 | 24.17 | 24.66 |  | | 1.04 | 0.8 | | 1.21 | |  | 95.9 | | 95.3 | 96.9 | |
| 1 | 25.51 | 25.09 | 25.68 |  | 23.13 | 22.28 | 23.76 |  | | 2.37 | 1.87 | | 3.03 | |  | 90.7 | | 88 | 92.7 | |
| ≥2 | 24.81 | 24.04 | 25.89 |  | 21.61 | 20.22 | 23.23 |  | | 3.21 | 2.41 | | 4.31 | |  | 87.1 | | 82.7 | 90.3 | |

*Notes*: * Proportion of life spent without 2 or more chronic health conditions between the ages of 50 and 75. ^†^ In SLOSH the presence of cardiovascular diseases were inquired with one item including both heart disease and stroke.
